# Preclinical models and evaluation criteria of prostatitis

**DOI:** 10.3389/fimmu.2023.1183895

**Published:** 2023-05-09

**Authors:** Hailan He, Hui Luo, Hui Xu, Biao Qian, Xiaofeng Zou, Guoxi Zhang, Fei Zeng, Junrong Zou

**Affiliations:** ^1^ The First Clinical College, Gannan Medical University, Ganzhou, Jiangxi, China; ^2^ Department of Urology, The First Affiliated Hospital of Gannan Medical University, Ganzhou, Jiangxi, China; ^3^ Institute of Urology, The First Affiliated Hospital of Gannan Medical University, Ganzhou, Jiangxi, China; ^4^ Department of Urology, Jiangxi Engineering Technology Research Center of Calculi Prevention, Ganzhou, Jiangxi, China

**Keywords:** prostatitis, chronic non-bacterial prostatitis, animal models, evaluation criteria, infectious prostatitis

## Abstract

Prostatitis is a common urological condition that affects almost half of all men at some point in their life. The prostate gland has a dense nerve supply that contributes to the production of fluid to nourish sperm and the mechanism to switch between urination and ejaculation. Prostatitis can cause frequent urination, pelvic pain, and even infertility. Long-term prostatitis increases the risk of prostate cancer and benign prostate hyperplasia. Chronic non-bacterial prostatitis presents a complex pathogenesis, which has challenged medical research. Experimental studies of prostatitis require appropriate preclinical models. This review aimed to summarize and compare preclinical models of prostatitis based on their methods, success rate, evaluation, and range of application. The objective of this study is to provide a comprehensive understanding of prostatitis and advance basic research.

## 1 Introduction

Prostatitis is an inflammatory disease of the prostate glands with a variety of complicated symptoms, including generalized pain in the pelvic and lower abdomen areas and obstructive and erectile dysfunction (1). Prostatitis is classified into four categories: acute bacterial prostatitis (I)), chronic bacterial prostatitis (II), chronic prostatitis/chronic pelvic pain syndrome (CP/CPPS)(III), and asymptomatic inflammatory prostatitis (IV) (**Table 1**). Type III CP/CPPS is further divided into IIIA and IIIB based on the presence or absence of leukocyte infiltration in prostate specimens (3). The prevalence of prostatitis is 8–25% in the urological surgery clinic, with Type III CP/CPPS being the most common, accounting for about 90–95% of cases (2, 4). Prostatitis, especially non-bacterial ones, is etiologically and pathogenetically complex. Prostatitis is associated with infectious and non-bacterial factors, such as spontaneousness, immune disorders, hormones, diet, stress, chemicals, urine reflux, exosomes, and autonomic nerves. There are several treatments for prostatitis, including antibiotics, painkillers, Alpha blockers, physiotherapy, prostate massage and lifestyle changes. These current treatments for prostatitis depend largely on the underlying cause and severity. Antibiotics fight bacterial infections; painkillers relieve pain and discomfort, and Alpha blockers relax the muscles around the prostate and relieve urinary symptoms. In the pelvic region, physiotherapy reduces pain and improves muscle function (5, 6). However, most treatments treat the symptoms but not the root cause, mainly because the mechanism is not clear. The causative factors of prostatitis are so diverse that it is essential to promptly choose the associated preclinical models from the various models to easily and efficiently investigate the condition. This review compared and summarized anatomy and histology of the human and rodent prostate, site of origin, function, symptoms, progression and immune system, as well as current preclinical models of prostatitis in method, successful rate, evaluation, and range of application.

**Table 1:** Categories of prostatitis.

Categories		Symptoms/analysis		Prevalence
Infectious prostatitis (I and II)		Acute or recurrent symptoms, with the same microgram		–
Chronic prostatitis/chronic pelvic pain syndrome (CP/CPPS) (III)	Inflammation (IIIA)	Leukocytes found in testing samples	No demonstrations of infection but recurrent symptoms	90%–95% (2)
	Non-inflammation (IIIB)	No leukocytes found in testing samples		
Asymptomatic inflammatory prostatitis(IV)		Leukocytes found in testing samples without related-symptoms		–

Symbol "-" means that there is no relevant literature to provide data.

## 2 Differences between men and rodents’ prostatitis

### 2.1 Prostate anatomy, histology and sites of prostatitis

The human prostate is a complex gland with diverse histological structures situated below the urinary bladder and anterior to the rectum. Comprising the prostatic urinary tract, the prostate gland encompasses the central zone (CZ), peripheral zone (PZ), and transitional zone (TZ), with the seminal vesicles located on both sides of the foundation. The CZ constitutes most of the base of the prostate and surrounds the ejaculatory ducts, while the PZ envelopes most of the central area and stretches caudally, partially enveloping the distal part of the urethra (7).

The glandular epithelium is composed of acinus and ducts lined with three types of cells, including luminal cells, basal cells, and neuroendocrine cells. Luminal cells are specialized cells that secrete a variety of products into the lumen, including prostate-specific antigen (PSA). Immunohistochemistry for PSA is strongly positive in luminal cells. The interstitium of the prostate is fibromuscular with abundant smooth muscle cells mixed with fibroblasts, blood vessels, and nerves. Notably, adipose tissue is absent in the prostate. This fibromuscular interstitium is much more pronounced than the comparatively thin fibromuscular interstitium found in the mouse prostate (8).

**Figure 1:**
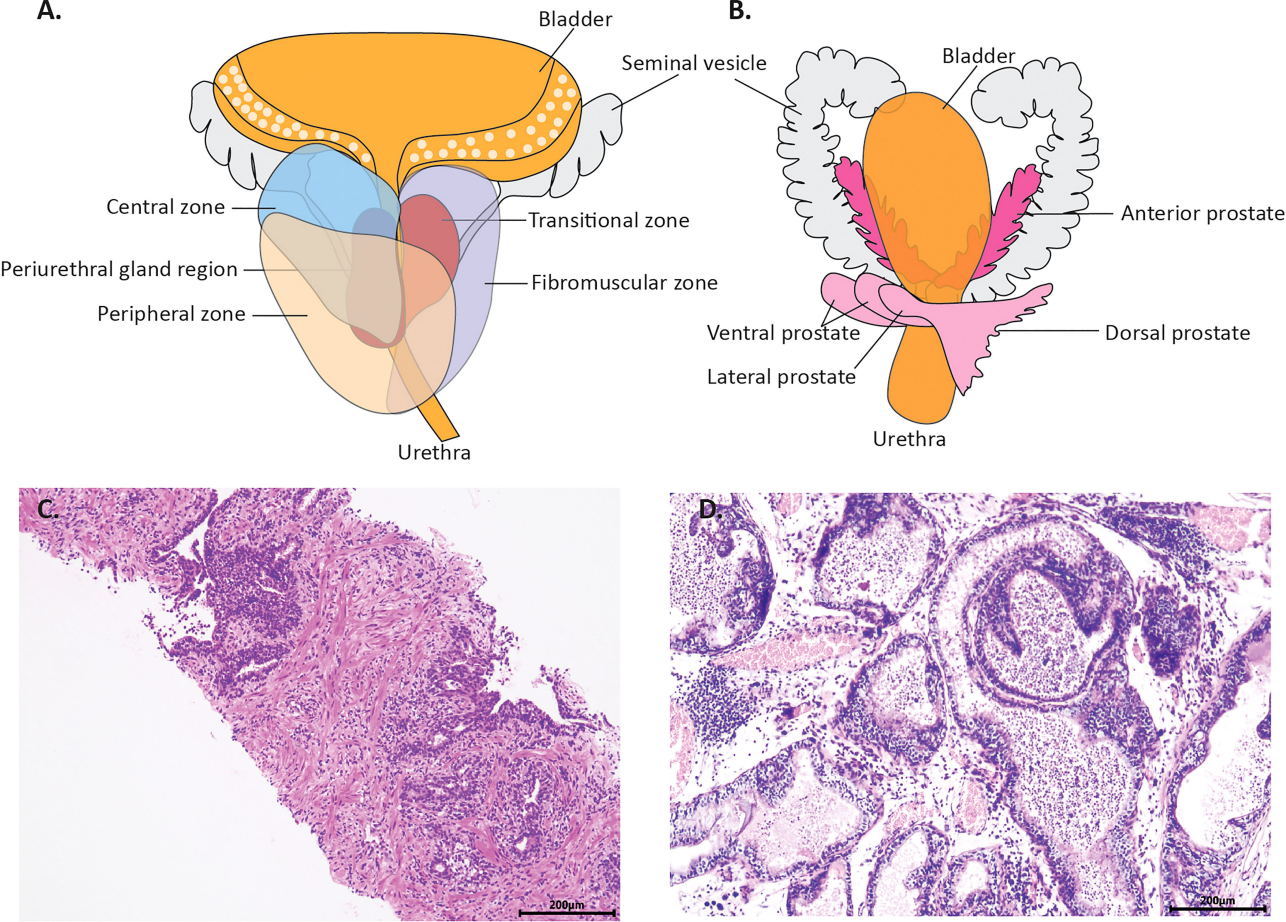
The anatomy of normal prostate and microscopic changes of prostatitis in a man and rodent. **(A)** is the schema human prostate. It consists of the central zone (CZ), the peripheral zone (PZ) and the transitional zone (TZ). **(B)** is the schema of rodents’ prostate. It consists of four distinct lobular structures: the anterior lobe the dorsal lobe, the ventral lobe and the lateral lobe, distributed on the left and right flanks. **(C)** shows the typical histology of prostatitis from a biopsy specimen, with inflammatory cell infiltration between the epithelial cells and in the interstitium. **(D)** shows the typical histology of prostatitis in a gonadectomized rats, with inflammatory cells in the interstitium and lumen.

In rodents, the prostate is also located below the bladder, enveloping the prostatic urethra, but it does not form a singular anatomical structure like in humans. Instead, the rodent prostate consists of four distinct lobular structures: the anterior lobe (also known as the coagulation gland), dorsal lobe, ventral lobe, and lateral lobe (**Figure 1**). The final appearance of each lobe is different due to differences in lobe-specific branching morphogenesis (9). The cytological distribution of rodent prostate tissue is of the identical type, but PSA is expressed and secreted in human, rather than mouse, intraluminal prostate cells. The most striking histological difference lies in the stromal component, which is highly developed as a pre-fibro-muscular region in humans, whereas in mice, it is sparse with very few smooth muscle cells. Moreover, the main histological features necessary for the different murine lobes vary (10).

Besides, the human PZ is a primary site of prostatitis and prostate cancer (7). The human prostate usually displays a variety of modifications in the epithelium and stroma. Atrophy of the epithelium and hyperplasia of the basal cells are extremely frequent. Chronic inflammation is also very prevalent, with varying degrees of acute inflammation seen, ranging from localized to widespread, forming abscesses (8). However, in rodents, the bulk of prostatitis occurs in the dorsal, ventral, and lateral lobes, with little inflammation occurring in the anterior ones. It has been shown that the proximal and distal regions of each prostate lobe in mice are most related to the ductal and acinar regions, respectively, and that the mouse lateral lobes are most closely related to the peripheral region in humans (11). Immune-mediated prostatitis models, hormone-disordered prostatitis models, and diet-related models appear to have dorsolateral/ventral lobe prostatitis in most models, similar to human prostatitis. This is consistent with the frequent occurrence of prostatitis in the peripheral zone, which facilitates the study of prostatitis. However, in the autonomic disorder model, prostatitis appears to occur predominantly in the anterior lobes.

### 2.2 Immune response during prostatitis

The immune response to antigenic stimulation in humans and rodents is broadly similar, involving cellular and humoral immunity (12). However, there are significant differences in the development, activation, and antigen response of the innate and adaptive immune systems in humans and rodents (13). The proportion of immune cells involved in inflammatory responses also varies, with neutrophils being more abundant than lymphocytes in humans, and lymphocytes being more dominant in mice (13). Notably, the rodent immune system is more similar to that of neonatal humans, with lower innate immune activation and naiver lymphocytes (14). While rodent models of prostatitis may not consistently reflect human immune system responses, they remain valuable tools for investigating the pathogenesis and treatment of the disease. Immunodeficient mice, in particular, have proven useful in a variety of medical research areas, including preclinical trials, regenerative medicine, transplant rejection studies, and immunotherapy (15).

Overall, current models of prostatitis have limitations. The anatomy, cytology, pathogenic zone, and disease pathogenesis of human and rodent prostate tissue differ, and rodent models fail to fully mimic the age of onset of human prostatitis. These limitations stem mainly from evolutionary differences between humans and rodents. However, the rodent prostate LP is most similar to the human peripheral zone, and immune-mediated, hormone-disordered, and diet-related models of prostatitis often show DLP/VP involvement, which are also affected in human prostatitis. This highlights the potential value of rodent models for studying the disease. This review focuses on currently reported models of prostatitis, including those involving specific bacterial infections, aging, autoimmune responses, hormonal disorders, dietary metabolic problems, stress-induced, chemical injury, exosome injection, mechanical introduction, and autonomic abnormalities. However, no definitive models of prostatitis caused by psychiatric, frequent sexual intercourse, pelvic floor dysfunction, or other factors have been reported in animal models.

## 3 Infectious prostatitis models

Infectious prostatitis is a disease caused by bacteria that infects the prostate gland. This inflammation has acute or chronic symptoms. The common pathogenesis is pathogens infection, including UPEC strain CP9 or C85, p. aeruginosa strain ATCC 27853, E. coli, or Chlamydia psittaci.

### 3.1 Acute bacterial prostatitis (I)

The prostate is predisposed to acute inflammation caused by bacterial infection. In rats and mice, pathogens damage tissues, causing acute inflammation, including UPEC strains *CP9* and *C85 (* 16), *p.aeruginosa* strain ATCC 27853 (17), *E. coli (* 18–22) or *Chlamydia psittaci (* 23). Acute bacterial prostatitis provides a better understanding of pathological changes, the location of pathogen entry, and the relationship between genetic background and bacterial invasion. Prostates can be infected by pathogens in two different ways. Mice can be injected with bacteria into their prostates (16, 18, 19, 23), or Wistar rats or Sprague-Dawley rats can be injected with pathogens through catheters into their prostates (17). During the first 18 days after infection, acute bacterial prostatitis causes lesions in the dorsolateral and ventral lobes, characterized by inflammation in the glandular lumens and interstitial tissue. After day 18, there was little evidence of inflammation (18). The intensity and concentration of pathogens determine the severity of acute prostatitis. Most bacteria damage the prostate of mice over 80%; specifically, *Chlamydia psittaci* and *E coli* infections cause 100% of mice to develop acute prostatitis (16, 22, 23).

### 3.2 Chronic bacterial prostatitis(II)

Prostatic damage caused by pathogens causes chronic bacterial prostatitis, typically taking 1–3 months longer. Pathology involves neutrophils and mononuclear cells infiltration in the interstitium or glandular cavity, as well as abnormal lesions of prostate tissue including occlusive changes and atrophy in acinus, epithelial hyperplasia and dysplasia. Chronic inflammation is challenging to cure, recurrent, and can be studied using mouse and rat chronic models for therapeutic research; after two months of exposure to *Escherichia coli Z17* (O2: K1: H-), 86.7% or 62% of Wistar rats developed chronic inflammation (24, 25), compared to 100% two weeks after *Chlamydia psittaci* infection (23). Two weeks after the onset of symptoms, Wistar rats displayed severe inflammation in the dorsolateral and ventral lobes, and 2–3 months later, they developed mild monocyte infiltration (23). C3H/HeOuJ mice manifested acute prostatitis with *E. coli* on the fifth day. It developed chronic inflammation 12 weeks later, characterized by reactive epithelial changes, including epithelial hyperplasia and dysplasia, as well as the level of Ki-67 increased which was known as a cellular marker for proliferation (21).

In acute and chronic inflammation, rodents are linked and differentiated from humans. In rodent bacterial prostatitis, there is a significant increase in neutrophils and macrophages in the interstitial, intraluminal or interepithelial cells of the prostate in the early acute phase, with T lymphocytes and macrophages infiltrating the tissue in the late acute stage (26). Yet, in humans, an acute inflammatory response occurs after the organism has recognized bacteria, stimulating the recruitment of neutrophils, monocytes and macrophages at the site of prostate infection (27, 28).

Upon development of chronic prostatitis, in the human prostate, the majority of infiltrating inflammatory cells are chronically activated T lymphocytes and macrophages (29, 30). Mouse models of inflammation show an infiltrative component closely resembling that of human prostate inflammation. A large number of lymphocytes infiltrate the mesenchyme, between the epithelium or in the lumen of the gland, including Th1, Th2 and Th17 cells (26, 31).

Prostatitis is usually caused by bacterial infection, and chronic bacterial prostatitis is often the result of prolonged acute infection. The lipid-like membrane barrier on the prostate surface is highly resistant to some therapeutic antibiotics, thus reducing desired medical concentrations and limiting potential therapeutic levels. Therefore, it is essential to develop models of bacterial prostatitis for research into a more effective treatment. While animal models are similar to humans regarding the clinical pattern of morbidity and histological lesion, they are predisposed to severe infection to death and remove bacteria so that chronic prostatitis is not quickly developed. Wistar or Sprague-Dawley rats are more susceptible to acute prostatitis (100%) than chronic prostatitis (50% or 62%). However, C3H/HeOuJ mice are more acceptable because they can develop chronic inflammation in the prostates and acute inflammation, and their success rate is 100%. These mice can be applied to the search for effective therapies. The advantages of cell models in noble medical analysis are that they are inexpensive, easy to access, and stable compared to mice. Cell models are also time-saving, successful, and can be used effectively for microscopic studies. However, these simple models do not represent the actual condition of complex multisystem patients. Optimization of animal and cell models should consider their limitations and maximize their benefits.

## 4 Non-bacterial prostatitis models

**Figure 2:**
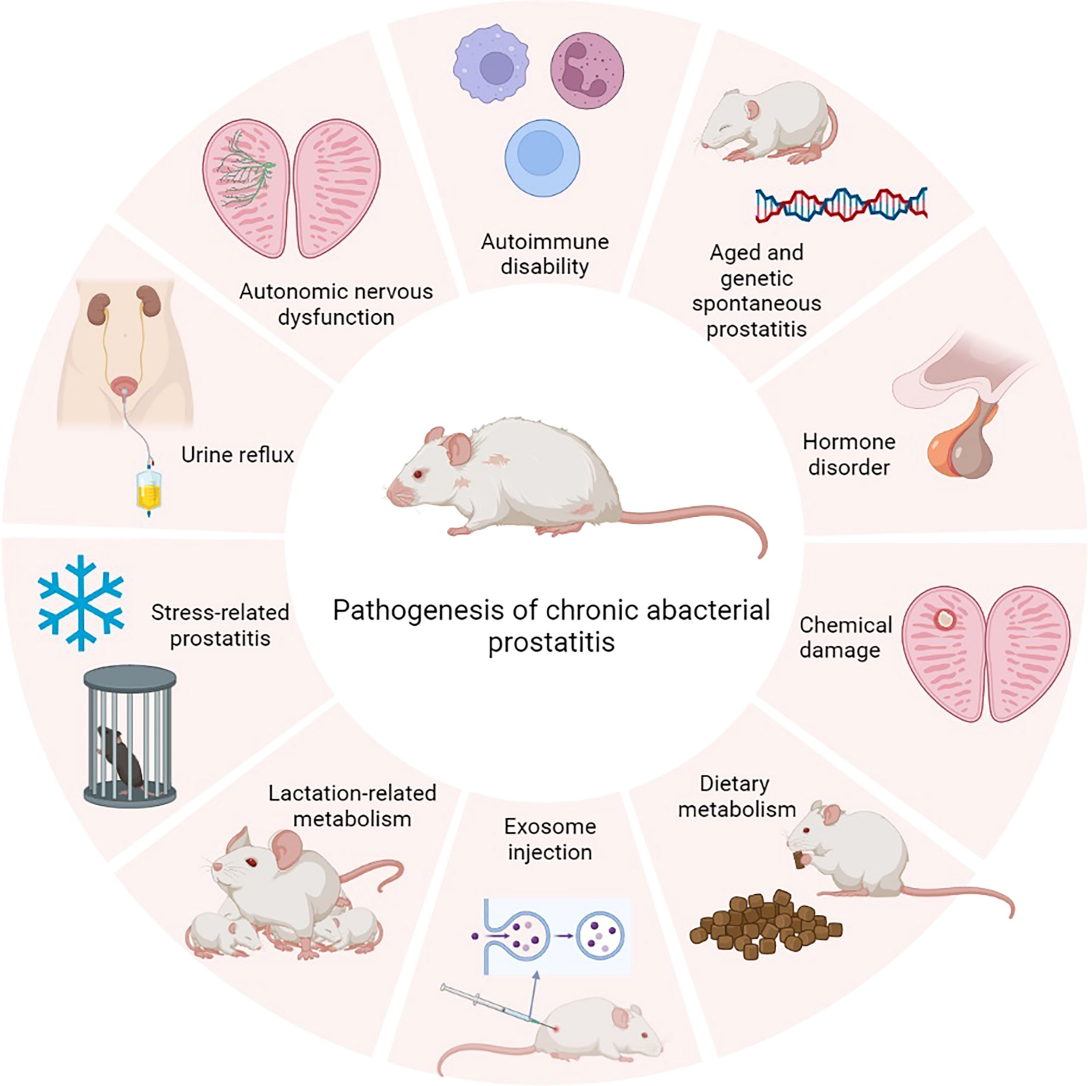
The pathogenesis of chronic prostatitis.

Chronic prostatitis has more complicated pathological changes than infectious prostatitis and is more obscure. Currently, there are several accepted animal models, such as spontaneousness, immune mediation, hormonal disorders, diet, lactation, stress, chemicals, urinal reflux, autonomic nervous dysfunction, and exosome (**Figure 2**).

### 4.1 Spontaneous prostatitis models

Rats and mice are predisposed to chronic non-bacterial prostatitis according to their genetic background and age in the absence of any treatment. This process takes a long time, but it is easy, and there are no medications or traumatic treatments. Inflammatory cells can be studied as part of the immune response by using this technique (32), the comparison of inflammation under different gene backgrounds (33, 34), different parts of inflammatory reaction in the prostate (33), the relationship between the occurrence and development of prostatitis and age (35, 36), oxidative stress response (37), and the role of specific genes in the occurrence and development of chronic prostatitis can also be explored. Rats of distinct types were more prone to spontaneous prostatitis than others Lewis rats are known to have an elevated susceptibility to the induction of many experimental inflammatory diseases such as inducible arthritis, glomerulonephritis, experimental myocarditis, autoimmune encephalitis, autoimmune thyroiditis and small bowel colitis (38). A total of 72% of Lewis rats developed spontaneous prostatitis by the age of 10–13 months (39, 40). In Wistar rats aged 10-13 months, 27% had spontaneous prostatitis (32, 39). After 13 weeks, 80% of older or younger developed spontaneous inflammation (41). In contrast to them, however, Sprague-Dawley rats are less sensitive to autoimmune related diseases. Only 16.6% of adult male Sprague-Dawley rats manifested prostatitis after a regular diet for 11 weeks (34). Noble rats of 9 months also developed prostatitis, but the success rate was uncertain (37).

Mice can be used to study prostatitis severity across genetic backgrounds. Spontaneous prostatitis occurred in Nonobese Diabetes-Resistant (NOR) and Nonobese Diabetic (NOD) mice at seven weeks of age, which became more severe and remained stable at 28 weeks (35, 36). They have a genetic susceptibility to autoimmune diabetes, and also have a tendency to other autoimmune diseases, including autoimmune thyroid disease and salpingitis (42). NZB (New Zealand Black, H-2d) mice are typically characterized by markedly receding thymus tissue, a functional defect in thymic epithelial cells compared to normal mice and an important autoimmune susceptibility factor (43). NZB strains also showed prostatitis at 14 weeks but were less sensitive and showed a lower inflammation score than NOD mice. At 18 weeks, prostatitis disappeared (36).

In summary, this spontaneous prostatitis occurred under natural conditions with fewer artificial factors and reduced artificial trauma. The models are stable and can be maintained for an extended period. This spontaneous non-bacterial prostatitis has a pathological picture similar to that of clinical patients and therefore is suitable for human chronic prostatitis research. Accordingly, it is more reliable than other models. Wistar rats can develop spontaneous prostatitis because they are more likely to succeed (80%), take less time to develop (13 weeks), and have a pathological picture closer to that of clinical patients. It is important to note that this model has numerous uncontrolled experimental factors and low targetability. The repeatability of this model is poor with prostatitis, as it takes three to thirteen months.

### 4.2 Immune-mediated prostatitis models

Autoimmunity is a double-edged sword. Too much strength or disorder can cause damage to the body’s tissues, including the prostate. Thymectomy, autoimmunity, and specific cell transplants can cause immune-mediated prostatitis, which was studied to determine the relationship between immune reaction and prostatitis pathogenesis.

#### 4.2.1 Thymectomy-related prostatitis models

**Figure 3:**
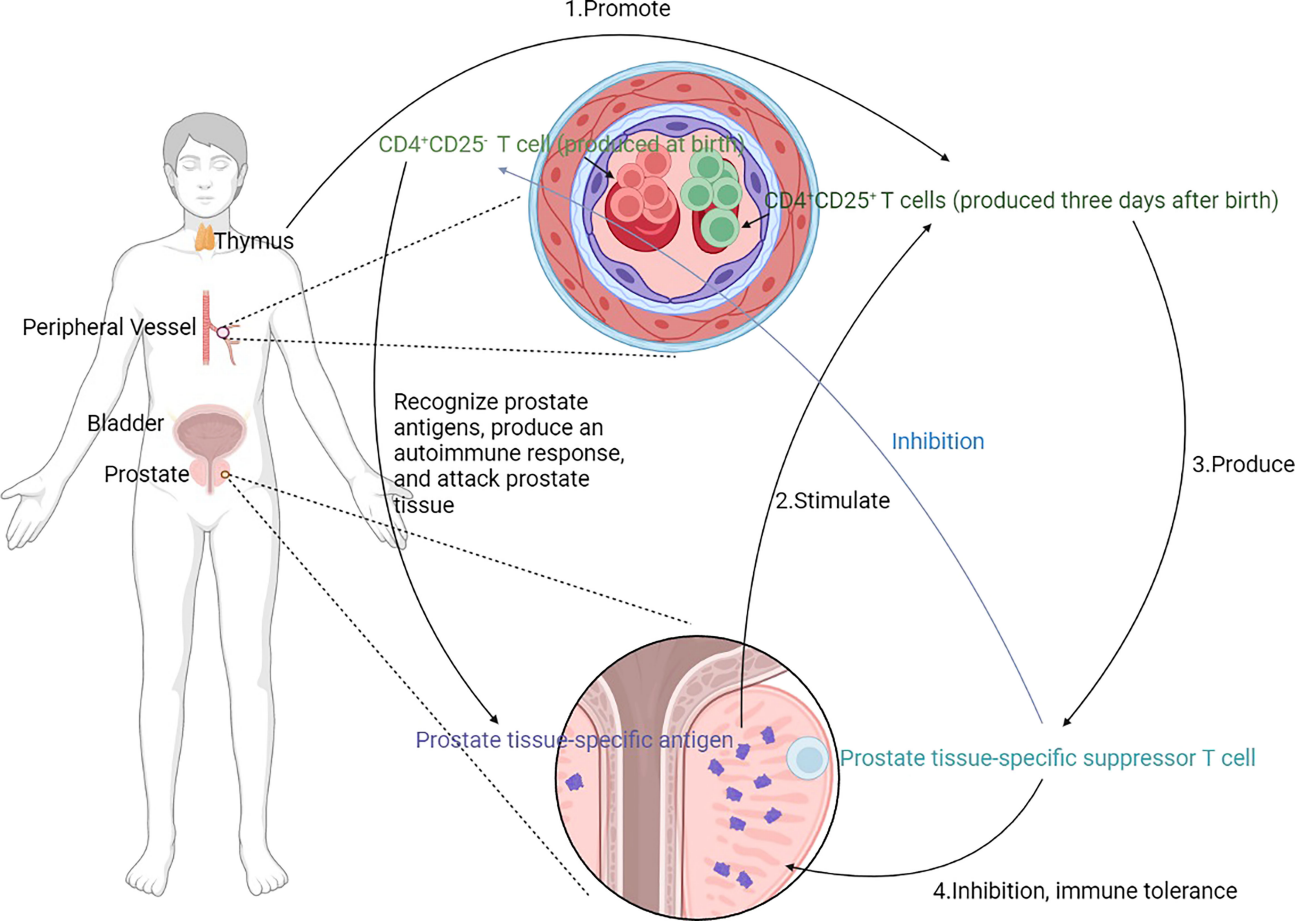
Mechanism of prostatitis induced by thymectomy. CD4+ T cells are crucial in the development of prostatitis. CD4+ CD25- T cells recognize prostate tissue-specific antigens and produce autoimmunity to attack prostate tissue. CD4+CD25+ T cells, on the other hand, inhibit this autoimmune response and only appear in the peripheral immune system 3 days after birth, stimulated by prostate tissue-specific antigens and promoted by the thymus. Prostate tissue-specific suppressor T cells are generated to suppress prostate tissue-specific antigens induced autoimmunity and produce immune tolerance. Moreover, the prostate tissue-specific suppressor T cells inhibit CD4+ CD25- T cells. Removal of the thymus at 3 days of life leads to an increase in CD4+CD25+ T cells, and a relative increase in CD4+CD25- T cells, which leads to an imbalance and the development of prostatitis.

The nonactivated suppressor T cells played a crucial role in preventing spontaneous prostatitis. CD4+CD25+ T cells appear as energetic regulators in the peripheral immune system at three days. After thymectomy for the postnatal three days, regulatory CD4+CD25+ T cells were significantly reduced, and spontaneous prostatitis occurred (**Figure 3**) (44–48). There is a correlation between autoimmune disorders and prostatitis that can be explored. Besides suffering from prostatitis after a thymectomy three days after birth, mice also suffered from inflammation in other organs after 150 days due to a systemic immune system disorder. A total of 73% (C3H/HeMs x 129/J) F1 developed prostatitis in this way, characterized by lymphocyte infiltration and parasecretion in epithelial cells within the anterior and dorsal lobes. The mice also developed gastritis, epididymitis, sialadenitis, and thyroiditis (44, 45). It was found that 58.6% of B6A mice also presented with lacrimal gland adenitis in addition to prostatitis (46). A total of 73% of NZM2328 mice had prostatitis along with thyroiditis and dacryoadenitis (48). Postnatal three-day thymectomy of SNF1 mice was associated with 61.5% prostatitis, orchitis, and aortitis (47).

This model provides an opportunity to study the basic principles of suppressor T cells, activated T cells, and androgen involved in self-tolerance. NZM2328 mice are more predisposed to prostatitis (73%) than other strains to investigate the autoimmune pathogenesis and function of various subpopulation T cells in prostatitis. However, it is complex and time-consuming to perform and administer a thymectomy due to the need for tracheal intubation and respiratory support. Furthermore, they have prostatitis and respond to other organs such as the testis, aorta, thyroid, lacrimal gland, and others.

#### 4.2.2 Experimental autoimmune prostatitis models

**Figure 4:**
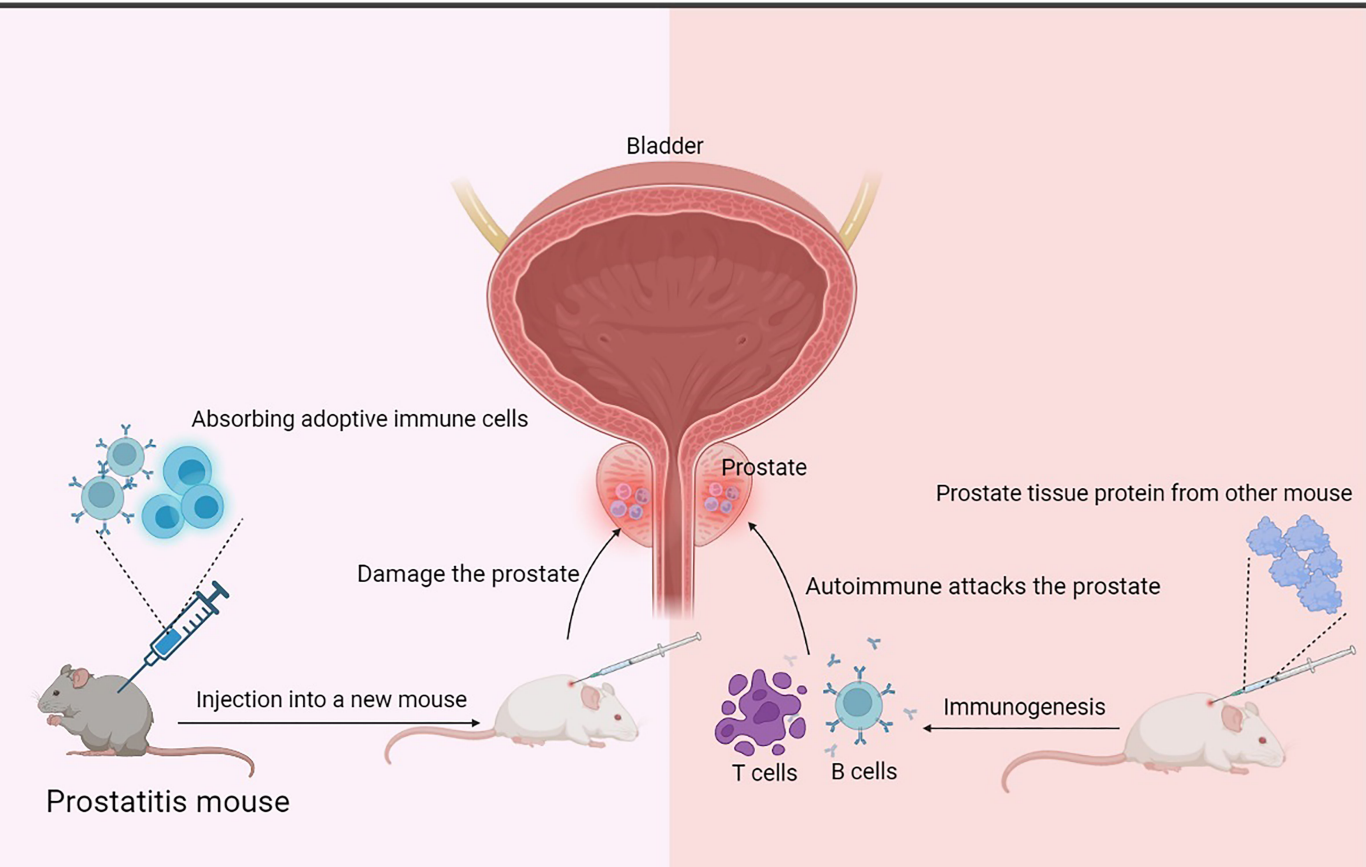
Mechanism of prostatitis induced by injection of prostatic antigen or adoptive immune cells. The principle of the EAP model is that proteins extracted from rat or mouse prostate tissue are injected subcutaneously to stimulate the mouse’s autoimmune response to produce inflammatory cells such as B cells and T cells associated with the injected prostate proteins, which migrate to the prostate tissue, infiltrate, and attack the prostate tissue, causing prostate damage and prostatitis. Cell transplantation-related prostatitis models are based on the principle that relevant immune cells are extracted from a model that has already produced prostatitis and injected into new mice, and that these adaptive immune cells may be specific enough to specifically travel to prostate tissue and attack it, causing damage and prostatitis.

Experimental autoimmune prostatitis (EAP) is a disease that can be viewed as an experimental model of non-bacterial prostatitis in humans. Using specific antigens to induce autoimmunity produces specific autoantibodies to attack prostate tissue (**Figure 4**). Therefore, the prostate tissue is subsequently damaged, resulting in prostatitis. Specific antigens include male accessory gland (MAG) homogenate (prostate, seminal vesicles, and coagulating glands) (35, 49–59), prostate extract (36, 60–63), peptide T2 (64–66), rat prostatic steroid-binding proteins(PSBP) (35, 59, 61, 67–69), prostatic acid phosphatase(PAP) (70), ethanol plus dinitrobenzene sulfonic acid (DNBS) (71, 72), MBP-SVS2 or MBP (73) or prostate-specific proteins p25 (74). To increase the likelihood of prostatitis, adjuvants that boost the immune response are often used, such as CFA, aluminum hydroxide adjuvant, and liposomes. It is important to note that antigenic immunity can occur within a species or among species. Prostate tissue from mice or rats can be extracted to induce autoimmune disorders in the same species of mice or rats (49–53, 60–63, 67, 69, 75), and prostate tissue from rats can also be used to induce other species of mice (35, 36, 56, 58, 59, 68). Both methods can induce prostatitis. Autoimmune reactions are one of the pathogenesis of chronic prostatitis that has been widely accepted and adopted. Furthermore, the procedure is relatively straightforward and requires two to three injections. There is less sensitivity in rats, with 38% of Wistar rats and 33% of Lewis rats presenting prostatitis.

Mice were more susceptible to EAP models than rats, including nonobese diabetic (NOD) (35, 36, 56, 58, 59, 68, 69, 76), C57BL/6 (56, 63, 65, 66, 72, 73), C57bl/6 lpr, SJL, AJ (63), NZB, SWR (36), BALB/c (36, 63, 69) and SWXJ (H-2q, s) (74). Most of these mice have autoimmune susceptibility. NOD mice have a genetic susceptibility to autoimmune diabetes and a predisposition to other autoimmune diseases, including autoimmune thyroid disease. C57bl/6 lpr mice develop within 3-6 moths a range of abnormalities of congenital disease, which describe the ‘lpr phenotype’ and permit them to progress to autoimmune abnormalities and multi-organ damage (77–79). Swiss James Lambert(SJL)mouse (80–82), A/J mouse (83), SWR mouse (84) and SWXJ (H-2q, s) mouse (85) have also been used for their increased susceptibility to autoimmune disease. The New Zealand Black (NZB) strain has the most similar clinical disease and genetic complexity to human disease (86, 87). BALB/c and C57Bl/6 mice also have autoimmune susceptibility because of a specific genetic background. The two mice have distinct structural and functional parameters of the immune system. secretion of IL-2, IL-3, IL-4, IL-10 and TNF-α is significantly higher in BALB/c mice than in C57Bl/6 mice. C57Bl/6 mice possess higher splenic NK cell inhibitory activity (88). Thus, injection of specific prostate tissue antigens triggers an autoimmune response in these mice, whereby the prostate tissue is assaulted by inflammatory cells, damage occurs, and prostatitis develops. These autoimmune diseases in mice often entail inflammation of multiple organs. The issue of what specific pathophysiological features of the prostate gland lead to prostatitis is not conclusive. However, it has been suggested that IgG4-related disease (IgG4-RD) manifests as autoimmune pancreatitis (AIP), but also as prostatitis. Histopathologically, there is a dense inflammatory infiltrate in the prostate interstitium and immunohistochemical morphometry shows 10 IgG4-positive plasma cells/high-power fields (HPF). There may be specific genes in the prostate that lead to inflammatory cell infiltration in the prostate tissue in autoimmunity, resulting in prostatitis (89). It has also been shown that prostate-specific antigen (PSA)-related autoimmune reactions develop, leading to the development of prostatitis (90).

C57BL/6 mice are commonly used for this model. The prostate supernatant was extracted from rats and emulsified with an equal quantity of complete Freund adjuvant (CFA), with a final antigen concentration diluted to more than 500 µg/ml. CFA is an immune adjuvant that enhances the immune response. It was challenging to induce autoimmune prostatitis with too low concentrations of prostate tissue extract, indicating that antigen concentration was the key to successful modeling. C57BL/6 mice have been subcutaneously injected into multiple sites with 0.5ml of this extract and 1 ng/200 µl Bordetella pertussis (BP) toxin. BP induced an autoimmune response in mice. After 30 days, all of them were perceived to have lymphocytic infiltration in the prostatic stroma, periglandular and perivascular areas (63).

The autoimmune disorder is a pathogenesis of chronic abacterial prostatitis that has been widely accepted and adopted. Treatment and immune changes are currently unclear and require more comprehensive studies in EAP models. C57BL/6 mice immunized with singular homologous prostate extracts are more likely to develop prostatitis than rats, with 100% showing signs of inflammation after 30 days. Nevertheless, this model had many disadvantages, such as the experiment always lasted 30-50 days, even 1-3 months, so the exact duration was not known; intricate factors affected the temporal consumption. The success of this experiment depended on the type of antigen, strain, and age of the rats, but also on the frequency, time, and parts of the inoculation. Due to 1–3 times immunizations and long or short interval time to establish prostatitis models, the method was complicated; the success rate differed from 33% to 100%.

#### 4.2.3 Cell transplantation-related prostatitis models

Cell transplantation-related prostatitis models are animals inoculated with isolated splenocytes from treated animals (immunization, hormonal treatment, or special gene background) (45, 73, 75, 91–93), GMTAMP-C1/C2 cells, TRAMP-C1/C2 cells (94), B7-TC1 cells (95) or T-cell receptor transgenic T-cells (91, 96–98). These splenocytes can attack the prostate tissue, leaving it damaged and inflamed (**Figure 4**).

The role of T and B cells in the pathogenesis of chronic prostatitis can be investigated using a cell transplantation model. It is also important to study and understand the function of immunocytes in chronic prostatitis. These observations suggest that immunological induction of prostatitis requires specific antigen organs, depletion of specific immune suppressive cells, or activation of suppressor cells. These models illustrate the basic principles of specific immunity, immune tolerance, and immune activity of chronic prostatitis. They also play an important role in researching the relationship between hormonal therapy and immunity, prostate cancer, and inflammation. To conduct research on the T cell response to specific antigens within prostatitis conveniently and proficiently, transgenic mice expressing prostate ovalbumin (POET) mice can generate a high level of membrane-bound ovalbumin/transferrin receptor fusion protein (mOVA) in prostate lobes under the action of ARR2PB promotors. Adaptive transfer with mOVA-specific T cells, will result in specific immunity (91, 96–98). This model can be applied to T cells that respond to specific organs of antigens. The POET mouse specifically contains mOVA expressed in prostate tissue but not in other organs. When the POET mouse is infused with mOVA-specific T cells, these T cells specifically bind to the mOVA expressed in the prostate and attack the corresponding area, resulting in tissue damage and prostatitis. This approach is designed to investigate the development and regulation of prostatitis, which occurs in the EAP but is also induced in other organs due to autoimmunity. However, the POET mouse allows inflammation to occur specifically in the prostate gland only by allowing specifically labelled T cells to attack prostate tissue directly. Furthermore, this model allows a clear and unambiguous view of the specific prostate lobe where inflammation occurs, ventral and dorsolateral lobes more sensitive to inflammation than anterior ones.

In addition, this POET mouse model can be applied to detect the effect of inflammation on the development and progression of prostate cancer. Because the duration of prostatitis in this model is longer compared to the EAP model, where inflammation lasts about 15 days, whereas the POET model lasts 70-80 days, it is beneficial to study the relationship between long-term stimulation of inflammation and the development of prostate cancer.

The Transgenic adenocarcinoma of the mouse prostate (TRAMP) model can also be studied for the association of inflammation with the development of prostate cancer. The GMTRAMP-C vaccine is derived from TRAMP epithelial cells and is tumorigenic. The GMTRAMP-C vaccine was administered to enhance the response of TRAMP to antigenic challenge, and TRAMP mice with strong antigenic challenge were then treated with anti CTLA-4, a protein on T cells that prevents T cells from killing other cells, including cancer cells, and anti-CTLA-4 Treatment causes T cells in TRAMP mice to be active and attack prostate tissue, causing damage and thus prostatitis. In addition to TRAMP mice, wild-type mice and non-transgenic C57BL/6 male mice sensitized with GMTRAMP-C1/C2 vaccine and treated with anti-CTLA-4 showed inflammatory infiltration of prostate tissue and even tissue damage including destruction of alveolar structures. Normal mice injected with GMTRAMP-C1/C2, a prostate tumor cell, promote immunogenetics, including T cells. T cells assault the prostate tissue, leading to structural destruction and prostatitis (94).

Adaptive transfer with spleen cells from female mice or castrated male mice at birth, C3.129 nu/nu mice are susceptible to prostatitis (45). The presence of specific antigens on the prostate tissue allows CD4+ CD25- T cells in the humoral fluid to recognize and induce autoimmunity on the one hand and promotes the production of prostate tissue-specific suppressor T cells to tolerate the immune response on the other (**Figure 3**). Female or castrated male mice at birth fail to produce prostate tissue-specific suppressor T cells to tolerate the immune response and instead have large numbers of CD4+ CD25- T cells, which are relayed to C3.129 nu/nu mice. C3.129 nu/nu mice have no thymus and are consequently immunodeficient, meaning that they are unable to mount an immune response to foreign substances. After relaying, CD4+ CD25- T cells recognize specific antigens on the prostate tissue of C3.129 nu/nu mice and induce autoimmunity to occur, which attacks the prostate tissue and damage occurs, resulting in prostatitis. It indicated that prostate tissue-specific suppressor T cells and nonactivated suppressor T cells play an important role in immune tolerance against prostate antigens.

#### 4.2.4 Knockout prostatitis models

*IFN-γ* -deficient NOD mice were perceived to significantly reduce the number of cell infiltration (35, 36). Interferon-γ (IFNγ) is an important mediator of the cellular immune response and is an inflammatory cytokine secreted by T lymphocytes and natural killer cells (NK cells), involved in the immune response *in vivo (* 99). Mice lacking IFN-γ expression had significantly less inflammatory cell infiltration in prostate tissue relative to controls in autoimmunity, indicating that IFN-γ -producing cells played a vital role in the development of infiltration in the prostate.

Autoimmune regulator (Aire) is essential for establishing central immune tolerance and preventing autoimmunity, and abnormalities or knockouts of *Aire* contribute to multi-organ autoimmune abnormalities in the body (100). It has been shown that *Aire-KO* mice inflamed multiple organs, in addition to inflammation in eyes, salivary glands, ovaries, stomach, and inflammatory cell infiltration in prostate tissue (101, 102). Autoimmune abnormalities resulting from *Aire* deficiency also contribute to inflammatory cells reaching, infiltrating, attacking and damaging prostate tissue, the exact mechanisms of which are unclear.

Taken these, thymectomy, autoimmunity, and specific cell transplants can cause the body to produce prostate tissue-related immune cells that attack and damage the prostate tissue, causing prostatitis. *IFN-γ*-deficient NOD mice and *Aire-KO* mice can be used as reproducible genetic models for studying the pathogenesis, prevention, control, and treatment of autoimmune prostatitis.

### 4.3 Gonadectomy and hormone-related prostatitis models

The relative level gives negative feedback to the hypothalamus dopamine and then promotes the pituitary gland to release prolactin, which activates specific receptors in the prostate glands to respond to androgen, thereby regulating the growth and development of the epithelium. However, by castration or hormone treatment, the hormone disorder induces a humoral and cell-mediated autoimmune reaction, the expression of inflammatory cytokines, or the destruction of the prostatic blood-lymph barrier to cause prostatitis and atrophy of prostate tissue. Hormone disorders are more likely to occur with age in humans. Therefore, hormone disorder treatment is generally accepted to establish models of abacterial prostatitis, including Hypo-gonadal (hpg) mice (103), (C3H/HeMs x 129/J)Fl mice (44, 46), Han-NMRI mice (104), Noble rats (105–107), Sprague-Dawley rats (108, 109), Lewis rats (39, 40) and Wistar rats (32, 39, 106, 110–115). Treatment methods include gonadectomy and 17β-estradiol injection, and one or both can achieve the model effect. Wistar rats were universally applied for this kind of model. This model was established within 14-36 days for some Wistar rats; for others, it took up to 18 weeks (106). Temporal consumption of this method depends on age, castration, and hormone treatment. Aged rats were more predisposed to prostatitis than young ones (32, 39, 111–113). Castration could shorten time based on hormone treatment (32, 111–115). In this model, inflammation appeared in ventral and dorsolateral lobes, while Aumüller G et al. discovered that all lobes had lesions after treatment with 17β-estradiol, testosterone, and castration in old rats (32). Furthermore, the lateral lobes manifested inflammation earlier than the dorsal lobes, and the ventral ones were the latest, characterized by neutrophil infiltration into the glandular lumen and plasma cells and lymphocytes infiltration into the interstitial area (110, 111). Wistar rats were treated daily with a total volume of 0.1ml 0.25 mg/kg 17β-estradiol for 30 days, and 100% of them demonstrated inflammation within lateral lobes, characterized with serious, multifocal, significant inflammation in a lobule, but in lateral prostates, there were no signs of inflammation (39). Different lobes of the prostate may have different sensitivities to hormonal disturbances.

Genetically modified Mice are available to research the development of prostatitis from specific genetic backgrounds. *AROM+* mice are laboratory mice that have been genetically modified to overexpress aromatase in the brain. Aromatase is an enzyme that converts testosterone to estrogen, and its overexpression in the brain leads to increased local estrogen levels, which can be used to study the connection between hormone disruption and prostatitis. Eighty-eight percent of *AROM+* mice were predisposed to prostatitis at 52 weeks. With androgen deficiency and rise in estrogen, multiple acute and chronic infiltration of inflammatory cells and increased chemokine and estrogen receptor alpha in prostatic epithelial cells resulted in prostatic intraepithelial neoplasia (116). Mice with different genetic backgrounds had different characteristics of prostatitis and affected the occurrence and development of prostatitis.

This model is reproducible, simple, inexpensive, and resembles the onset and persistence of chronic non-bacterial prostatitis in humans. The etiology of this disease may be similar to that of humans, which usually develops after birth when androgen levels are disturbed. Many models are evolving mice and rats, but the latter are more susceptible. Wistar rats are helpful models, as they are only treated with 17-estradiol, have a high probability of success, are reasonably straightforward to operate, and need less time to develop lesions. Both hormonal injections and castration are used in the model established by numerous researchers. However, castrated operation requires advanced aseptic techniques, and it is complex to perform and very difficult to perform on neonatal rats in some research. *AROM+* mice can be used as reproducible genetic disease models for studying the pathogenesis, prevention, control, and treatment of hormone-related prostatitis.

Furthermore, model phenotypes are often significantly correlated with estrogen dosage, time of exposure, and presence of androgens, which means that modeling results are unstable. Various ratios of estradiol may play an important role in inflammation. Additionally, high doses of medicine injection may result in toxicity, hepatic injury, and endocrine disruption.

### 4.4 Diet and lactation-related prostatitis models

Models of diet-related prostatitis involve rats or mice that exhibit prostatitis through simple feeding rather than injection or surgery, evolving diet soy-free (34), soy-extracted genistein with daidzein-rich Isoflavone (117), high fat (118–121) and lipid (122). Several studies have been conducted on the relationship between metabolism and prostatitis. Sprague-Dawley rats demonstrated prostatitis with a soy-free diet after 11 weeks (34). Following oral administration of genistein and daidzein-rich isoflavone extracted from soy, almost 83% of these rats developed dorsolateral prostatitis. During histopathological examinations, many neutrophils and lymphocytes were found to invade the stroma and glandular lumen, but body weight, prostate weight, and serous androgen levels did not change (117). High-fat diet(HFD) for 1–3 months, and even at seven months, established prostatitis models successfully, including Sprague-Dawley rat (119), NF-κB-Luciferase transgenic mice (121), aged FVB mice (118), and C57BL/6 mice (120). With HFD for 4 weeks, C57BL/6 mice presented different degrees of lymphocyte aggregates in the dorsolateral lobes and a significant increase in IL-1β, IL-6, IL-17, and TNFα (120). Lipid diet also caused prostatitis and was associated with mice with different genetic backgrounds. C57/BL6 mice had smaller prostates, a wider lumen, and shorter epithelium with linseed oil in the ventral lobes while increasing epithelial volume with soybean oil and inflammatory infiltration with pork fat for 32 weeks. Pork fat, conversely, caused Mongolian gerbils to develop larger epithelial lesions and larger inflammatory foci, whereas linseed and soybean oil caused a reverse effect (122). With or without treatment, the majority of newborns displayed prostatitis at the age of 90 or 120 days after lactation for 25 or 56 days from dams fed at the time of gestation(GD) or neonatal period for several days with different substances, including methoxychlor, pimozide (123, 124), atrazine, bromocriptine (125), tamoxifen, 17β-estradiol (123), estradiol benzoate (108), high fat diet (119) or vinclozolin (126–128). The role of lactating mothers with abnormal metabolic levels in the occurrence and development of neonatal prostatitis can be explored. Wistar rats were predisposed to inflammation and fibrotic alteration in the ventral and lateral lobes after perinatal exposure to methoxychlor, pimozide, trazine, 17-estradiol, or bromocriptine (123–125). Sprague-Dawley rats presented prostatitis with lactation from the administration of estradiol benzoate administration (108), a high-fat diet (119), and vinclozolin (126–128). Vinclozolin is a widely used dicarboxamide fungicide whose primary action is due to its competitive androgen receptor antagonism and antiandrogenic activity. Females were administered orally daily on GD14–GD19 with 100 mg/kg vinclozolin. On postnatal day 56, all newborns developed prostatitis, with significant and focal areas of infiltration of inflammatory cells into the ducts and blood vessels, especially leukocytes and macrophages, degenerate epithelial cells, decayed duct structures, and reduced epithelial cells androgen receptors and decreased secretory epithelium (128).

In summary, oral treatment is more straightforward and inexpensive than parenteral administration. Compared to inoculation, the genistein diet is safer because it is challenging to ensure a suitable injection dosage. A high dose of genistein inoculation may cause drug toxicity, hepatic injury, and endocrine disorder. Thus, Sprague-Dawley rats effectively prevent prostatitis by taking oral isoflavones rich in genistein and daidzein extracted from soy. Metabolic disease is a constant concern, and a high-fat diet can help prevent prostatitis. C57BL/6 mice are perceived to have visible prostatitis with HFD for four weeks because they can quickly develop into severe obesity, fat accumulation, and impaired glucose tolerance to appear prostatitis. To investigate the effects of maternal hormone on newborns, vinclozolin is widely used for the induction of prostatitis. Lactation of dams taking this medicine reverted prostatitis in 100% of Sprague-Dawley rats.

Furthermore, vinclozolin does not cause maternal toxicity, or normal pregnancy. The disadvantages of these prostatitis models are that they require a longer experimental period and are influenced by variations in the ratio of diet, the type of lipids and the form of the diet. It is also essential to ensure the reproducibility and similarity of the nutrient composition for a long time. These models are affected by many other factors, including strains, age, and time. The treatment of lactation and natural mating can be complicated by the intrauterine environment, breastfeeding, parent handling, and behavioral differences.

### 4.5 Multifarious prostatitis models

Stress, chemicals, exosome injections, mechanical operation of urine reflux, and autonomic nervous blockers induce chronic prostatitis models.

#### 4.5.1 Stress-related prostatitis models

The mechanism of stress induced prostatitis may be a change in the internal environment or an undiscovered mechanism needed to be further explored. When Sprague-Dawley rats were treated with stress conditions, such as starvation, 4°C temperature, and narrow cages, they developed prostatitis after ten days. Histopathological analysis in rats found an embedded acinus in the prostate, which contained active prostate epithelial stroma with less secretion. More inflammatory cells were in the acinus and stroma and a greater density of acinus and stroma. However, the foci and many inflammatory cells in the prostate prevented accurate classification (129, 130).

#### 4.5.2 Chemical-related prostatitis models

Chronic prostatitis was produced by direct chemical injection, including λ-carrageenan (131–133), capsaicin (134–136), and doxycycline (40). This method was assigned to help study the neurobiological mechanisms of male pelvic pain, although it caused damage, destruction, or degeneration of the prostates. A high dosage of chemical treatment could cause stress reactions and even death in rats. Hence, controlling time, dose, and injection parts was critical. Chen CS et al. elucidated that 100% of Sprague-Dawley rats were perceived to have prostatitis in the ventral lobes after one day and maintained the two weeks with 3% λ-carrageenan in accordance with thermal hyperalgesia, mechanical allodynia, inflammatory cell count, COX2 expression and Evans blue (131–133). In this study, a high level of inflammatory cell infiltration was detected, such as monocytes, lymphocytes, fibrous connective tissue hyperplasia, interstitial congestion, and edema, in addition to other chronic inflammatory conditions. Furthermore, inoculation with doxycycline caused 100% of Lewis rats to develop granulocytes in the lumen and inflammation in the stroma (40).

#### 4.5.3 Exosome-related prostatitis models

Exosomes are membrane-bound extracellular vesicles produced in the intranuclear region in most eukaryotic cells. They comprise a wide range of biomolecules, including proteins, lipids and nucleic acids such as RNA and DNA. Exosomes are involved in intercellular communication and play a role in a variety of physiological and pathological processes. They are secreted by a wide range of cells, including immune cells. Exosomes secreted by immune cells can stimulate an immune response (137). There are not currently many studies on exosome and prostatitis. Baixiong Zhao et al. established a noble prostatitis model with exosome injections (138). They extracted exosomes from prostatic fluid samples of CP/CPPS patients and then injected them into the ventral lobes of Sprague-Dawley rats. After one-week, numerous inflammatory cells infiltrated interstitial tissue because these exosomes were selectively loaded with miRNA-155 and heavily phagocytosed by prostate stromal cells to activate the immune response and induce inflammation. This model may be a novel method for studying the pathogenesis of chronic prostatitis.

#### 4.5.4 Urine reflux-related prostatitis models

Rats were artificially assigned to prostatitis through urine reflux to study another pathogenesis of chronic prostatitis. They were perfused with urine through the urethral orifice, the interstitial area was significantly enlarged, and polymorphic cells were diffusely distributed in the prostate after seven days. The acini did not contain inflammatory cells like the bacterial prostatitis model. Moreover, no effect was observed in the bladder due to histological changes. Inflammation-related proteins (IL-1A, IL-1B, IL-6, and TNFa) and oxidative stress markers (MDA and HIF-1A) increased in this model (139).

#### 4.5.5 Autonomic nervous dysfunction-related prostatitis models

The autonomic nervous system and the immune system are closely linked, and they communicate with each other through various pathways. For example, the sympathetic nervous system can regulate the activity of immune cells (such as T cells and natural killer cells) through the release of neurotransmitters such as norepinephrine and epinephrine (140). Likewise, immune cells generate cytokines and other signaling molecules to influence the activity of the Autonomic nervous system, and abnormal regulation of the Autonomic nervous system is relevant to a variety of immune-related disorders such as autoimmune diseases, chronic inflammation and allergies (141). Notably, the autonomic nervous system is involved in the occurrence and development of prostatitis. C57BL/6 mice received 5 mg/kg body weight of 1-adrenergic or 2-adrenergic receptor agonists intraperitoneally for five days and developed chronic prostatitis, characterized by increased pro-inflammatory cytokines TNF, IL-6, and chemokines CCL2, CCL3. After blocking the sympathetic and parasympathetic nerves, the mice developed persistent chronic prostatitis. This is a phenomenon, the detailed mechanisms of which are not yet known. It suggests, nevertheless, that there is a correlation between abnormal autonomic nerves and the development of prostatitis. Furthermore, adrenoceptor Beta 2 (Adrb2) and the accumulation of CD11b + F4/80 + macrophages were highly expressed in the prostate without sympathetic nerves. Chronic prostatitis can be studied by examining autonomic nervous and immune responses (142).

In summary, stress and urine treatment can be the pathogenesis of prostatitis, and it took less time than other models, just 7-9 days, which is advantageous for establishing this type of model. The autonomic nervous dysfunctional model related to exosomes may be novel methods for studying the pathogenesis of chronic prostatitis. The advantages of chemical treatment are simplicity, cost-effectiveness, and reproducibility, and it has strengths in research on gene-environment interactions and chemoprevention. This model tends to self-medicate and does not follow a chronic course but can cause severe lesions if administered in a high dosage of chemicals. Attention must be paid to the batch of chemicals, the breed, and source of the animal, the chemical supplier, dosage, frequency, and duration. Cellular models present advantages such as stability, high success rates, and savings of time and money. We can study microscopic examination in cells, but they are very different from the condition and consist of multiple treatment systems. It is possible to pre-estimate novel treatments in cellular models with efficient and time-saving outcomes before animal experiments.

## 5 Evaluation criteria

Pathological changes, biochemical analysis, behavioral testing, cutaneous allodynia evaluation, body weights or prostate, and urodynamic measurements can be used to diagnose the success of the prostatitis model.

### 5.1 Pathological changes

Pathology, including macro- and microscopic analysis, is critical for detecting prostatitis in animals. Macroscopic analysis is intuitive, simple, and convenient for grossly morphologically estimate the situation of inflammation within the prostates. After dissection, gross morphological analysis of infected prostates is observed for obvious signs of inflammation, including edema, congestion, and hyperemia (16, 71, 72).

Microscopic changes are more detailed and accurate than macroscopic changes, including acute and chronic prostatitis. In acute prostatitis, the dense infiltrate of acute inflammatory cells (neutrophils) in the periglandular stroma, interstitial edema, focal hemorrhage, and abundant shedding of epithelial cells into the lumen of the prostatic gland of acute prostatic tissue are revealed by the microscope (21). In chronic prostatitis, changes in the lesion were scored based on three histological conditions: edema, hemorrhage, and infiltration of leukocytes. This method depends on the subjectivity of observers by comparing the severity of appearance and diving into four grades (16, 143, 144).

### 5.2 Biochemical analysis

Prostatitis is characterized by an aberrant immune response and a cascade of inflammatory cytokines. These cytokines, such as interleukin-1, interleukin-6, interleukin-8 and tumor necrosis factor-alpha (TNF-alpha), play a critical role in the modulation and amplification of the immune response, as well as the propagation of inflammation. In the context of prostatitis, the overproduction of these cytokines by immune cells is closely associated with the development of the inflammatory response. Therefore, the measurement of cytokine expression, particularly IL-1, IL-6, IL-8 and TNF-α, provides a reliable indicator of the success of the prostatitis model (19, 61, 64, 68, 76, 145).

Prostate inflammation also involves various molecules and signaling pathways. Among these are the C-C motif chemokine ligand (CCL) family, a group of chemokines that play crucial roles in immune responses and inflammation. The CCL family encompasses a range of chemokines, including CCL2, CCL3, CCL4, and CCL5, all of which contribute to the development of inflammatory responses. In chronic bacterial prostatitis, there is a significant increase in the expression of CCL2 and CCL3 in prostate tissue. This upregulation of CCL2 and CCL3 is known to promote the recruitment of immune cells to the site of inflammation, thereby exacerbating the inflammatory response. Moreover, recent studies have identified CCL2 and CCL3 as significant biomarkers for inflammatory IIIA and non-inflammatory IIIB chronic pelvic pain symptoms, underscoring their potential clinical significance in the diagnosis and management of prostatitis (146).

In general, tests for IL-1, IL-6, IL-8 and TNF-α expression levels can be an indicator to verify the success of prostatitis. CCL2 and CCL3 expression levels have been found to be altered in prostatitis, although they are not necessarily markers.

### 5.3 Multifarious analysis

Behavioral testing, evaluation of cutaneous allodynia, body or prostate, and urodynamic measurements can be used to diagnose the success of the prostatitis model. Behavioral testing is often used in the evolution of pain assessment (57, 69, 74) and cutaneous allodynia assessment of chronic pelvic pain (76, 112). Both can be used to test for the pain of chronic prostatitis. Weights of the body or prostate can be used to determine the changes in prostate tissue before and after treatment (74, 106, 108, 113, 117). Prostatitis causes changes in urodynamic measurements, elucidating that prostatitis models reduced the frequency of urination and the prolonged urination time, and the residual urine volume of hormone-treated rats tended to increase (106, 107, 112).

## 6 Conclusion and prospects

Prostatitis is a common condition observed in the urology department, with a high prevalence. Despite the complexity and uncertainty of the pathogenesis and etiology, effective treatment is imperative, so it is crucial to choose the appropriate models quickly and conveniently. Based on the models of prostatitis mechanically described above, different settlements have different manufacturing methods, durations, inflammatory lobes, success rates, disadvantages, and evaluation criteria (**Table 2**). The search for more suitable models to explain the etiology, pathogenesis, and clinical manifestations of prostatitis should continue in the future.

**Table 2:** Summary of present prostatitis models.

Categories		Model	Treatment	Duration	Lesion sides	Advantages	Disadvantages	Success rate	Recommend	References
Infectious prostatitis models	Acute bacterial prostatitis	RWPE-1 cell	AIEC strains, lipoteichoic acid, Trichomonas vaginalis	2, 3, 24 hours	/	Inexpensive, easily accessible, stable, high success, microscopic studies, earlier, saving time and money.	Considerable difference from actual conditions of patients.	/	/	(147–150)
		Rat: Wistar, Sprague-Dawley	, *E. coli*, Chlamydia psittaci	0-18 days	Dorsolateral, ventral lobes	High incidence, morbidity and histological lesion similar with human,	Predisposal to severe infection to death, self-healing at short time	83.3%-100% (16, 22, 23)	Sprague-Dawley rats C3H/HeOuJ mice (16, 21–23)	(16–23)
		Mouse:C3H/HeJ	E. coli	5 days				100% (20)		
	Chronic bacterial prostatitis	Rat: Wistar, Sprague-Dawley	Escherichia coli, Chlamydia psittaci	1-3 months			Less sensitive than acute prostatitis, bacterial infection predisposal to recovery and difficult to chronic inflammation.	50% (22), 62% (24), 86.7% (25).	C3H/HeOuJ mice (21)	(21–25)
		C3H/HeOuJ mouse	E. coil	12 weeks				100%		
Spontaneous prostatitis model		Rat: Lewis, Wistar, Sprague-Dawley	/	3-13 months	Dorsolateral, ventral lobes	Fewer artificial factors, less artificial trauma, stable, long period of time, similar to the clinical patients, age-related, specific genetic	Full with uncontrolled experimental factors and low targetability, spending a long period in prostatitis, ranging from 3-13 months, poor repeatability.	Lewis:30% -72% (39, 40); Wistar:27% -80% (32, 39, 41); Sprague-Dawley:16.6% diet for 11 weeks (34).	Wistar rats (41)	(32–37, 39–41, 43)
		Mouse: NOD, NZB	/	7-28 or 40 weeks	Ventral, dorsal or whole lobes			/	NOD, NZB (35, 36, 43)	
Immune-mediated prostatitis models	Thymectomy-related	Mouse:(C3H/HeMs x 129/J) F1, B6A, SNF1, NZM2328	Thymectomy of postnatal 3 days	40-150 days	Anterior, dorsal, ventral(infrequency)lobes	Self-tolerance, such as suppressor T cells and activated T cells from thymus.	Difficult to thymectomy, multiple inflammation	(C3H/HeMs x 129/J)F1: 27%-73% of prostatitis (44, 45); B6A:58.6% of prostatitis (46); SNF1: 61.5% of prostatitis (47) NZM2328: 73% of prostatitis (48).	NZM2328 mice (48)	(44–48)
	EAP -related prostatitis models	Rats: Wistar, Lewis, Sprague-Dawley, Copenhagen	Antigen,1-3 times of immunizations.	12 hours, 30-50 days, 1-3 months	Dorsal, ventral and lateral lobes	High clinical similarity, dose-dependent manner, genetic background, different sensitivity	Depend on operating enviroment, volume and purity of antigen, the frequency, time and parts of inoculation	Wistar: 38% (49) Lewis:33% of prostatitis (75) Sprague-Dawley:100% (64, 71) Copenhagen:80% (70)	C57BL/6 mice (63)	(35, 36, 49–53, 56, 58, 59, 61–66, 68–76)
		Mouse: NOD, C57BL/6, SJL, AJ, BALB/c, NZB, SWR		2, 10-42 or 63 days	All	NOD:100% (56, 59); C57BL/6: 37.5%-100% (56, 63, 65, 66, 73) SWXJ (H-2q, s):100% (74).				
	Cells injection-related prostatitis models	Rat: Lewis, Wistar	Splenocytes	7,10,30 days	Dorsal and ventral	The pathogenesis and function of immunocytes of chronic prostatitis can be examined	Limit and difficult to take a comprehensive acknowledge of development of prostatitis.	62.5% (75); 90% (93),100% (92)	POET (91, 96–98),C3.129mice (45), POET-3mice (91),TRAMP mice (94).	(45, 73, 75, 91–93, 95, 96)
		Mouse: C57BL/6, POET1/3,		About 60-90 days	ventral anterior dorsolateral	*C3.129 nu/nu*:more than 90% (45) *RAG-KO B6:*100% *(* 73) C57BL/6: 100% (95) POET1/3:100% (96)				
	Knockout prostatitis models	Mouse: IFN-γ -deficient NOD, Aire-KO	Knockout	/	Ventral, dorsolateral lobes	The ability to look specifically at the mechanisms by which prostatitis develops, down to specific molecules or genes.	Basic life activities are severely affected and feeding is difficult.	Aire-KO:71% prostatitis (73).	IFN-γ -deficient NOD (35), Aire-KO:71% prostatitis (73).	(35, 73)
Gonadectomy and hormone-related prostatitis models		Rat: Lewis, Wistar, Noble, Sprague-Dawley	Orchiectomized, 17β-estradiol	2-6 weeks, 10-18 weeks	Ventral and dorsolateral lobes	Reproducible, simple, inexpensive, a resemblance to the onset and persistence of chronic nonbacterial prostatitis in humans, the etiology of this disease be similar to humans.	Complex to to perform on neonatal rats, drug toxicity, hepatic injury and endocrine disorder.	Lewis:100% (39, 40) Wistar:100%of prostatitis (32, 39, 111–114)	Wistar rats (39)	(32, 39, 40, 44, 46, 103–114, 116)
		Mouse: *hpg*, Han-NMRI, AROM+	17β-estradiol, castration	42 or 90 days	Anterior, dorsolateral and ventral lobes			(C3H/HeMs x 129/J) F1: 54% (44) Han-NMRI: 100% (104). AROM+: 21%-88% prostatitis; 33% PIN lesion; 60% scrotal hernias (116)		
Diet/lactation-related prostatitis models	Diet-related	Sprague-Dawley rat	soy-free, genistein, high fat	9-11,26-29 weeks	Dorsolateral lobes	Simple and inexpensive, to explore the effect of maternal hormonal on newborns, C57BL/6 mice can easily develop into severe obesity, fat accumulation and impaired glucose tolerance to appear prostatitis	Longer experimental period, influence by various elements, variation in the ratio of high fat diet, intrauterine environment, breastfeeding, parental handling, and behavioral differences	80% (34);83% (117) /	Sprague-Dawley rats (117, 128), C57BL/6 mice (120).	(34, 108, 117–128)
		Mouse:C57/BL6		4-9, 12, 32 weeks	Ventral dorsolateral lobe					
	Lactation-related	Rat: Wistar, Sprague-Dawley	17β-estradiol fat diet vinclozolin	12-15 weeks	Ventral dorsolateral lobes			Methoxychlor:60% (123); Tamoxifen: 27.89% (123); 17β-estradiol: 90% (123); Estradiol:20% (124); Vinclozolin:100% (126–128)		
Multifarious prostatitis models	Stress-related	Sprague-Dawley rat	Starvation/4 °C temperature/narrow cages	10 days	/	Simplicity, cost-effectiveness, reproducibility, strengths in research on gene-environment interactions and chemoprevention;	Chemical-related models are self-medication, not to follow a chronic course, severe lesion if a high dosage of chemicals, influence by the batch of chemicals, the breed, sex and source of the animal, the chemical supplier, dosage, frequency and duration. The cellular models are very different from the actual condition consist of multiple systems regulation of human.	/	Sprague-Dawley rats (129–133, 138, 139)	(40, 55, 129–133, 138, 139, 151–154)
	Chemical-related	Rat: Sprague-Dawley, Lewis	3%λ-carrageenan/capsaicin/doxycycline	1 day	Ventral lobe			Sprague-Dawley:100% (132) Lewis:100% (40)		
	Exosome-related	Sprague-Dawley rat	Exosome from prostatic fluid	7 days	Ventral lobe	Novel research direction about exosome		/		
	Urine reflux-related	Sprague-Dawley rat	urine	7 days	/	Novel research direction about urine reflux		/		
	Autonomic nervous dysfunction-related	C57BL/6 mice	β2-adrenergic receptor agonists	5 days	Anterior lobe	Study of relationship between autonomic nerve and prostatitis		/	C57BL/6 mice (142)	

## Author contributions

JZ and HH designed the manuscript and outline for the review. HH, HL, and HX searched related publications. HH and FZ drafted the manuscript. JZ, XZ, and GZ reviewed the manuscript and polished the grammar. All authors contributed to the manuscript revision and approved the submitted version.
